# ‘Austria First’ revisited: a diachronic cross-sectional analysis of the gender and body politics of the extreme right

**DOI:** 10.1080/0031322X.2019.1595392

**Published:** 2019-05-23

**Authors:** Markus Rheindorf, Ruth Wodak

**Keywords:** Andreas Gabalier, anthem, banal nationalism, body politics, Discourse Historical Approach, election campaign, extreme right, FPÖ, gender politics, Johann Gudenus

## Abstract

In this paper, Rheindorf and Wodak provide a discourse-historical analysis of extreme-right cultural politics in Austria, ranging from the blatant racism in the speeches of Vienna’s former Deputy Mayor Johann Gudenus (now MP in the Austrian parliament) to the construction of an idealized national body in the election campaigns of the Freiheitliche Partei Österreichs (FPÖ), its programmatic agenda in handbooks and pamphlets, and the performances of far-right pop singer Andreas Gabalier. Rheindorf and Wodak argue that such cultural politics use a wide spectrum of discursive strategies both inside and outside established party politics and that the accompanying production of an ideal extreme-right subject is informed by nativist ideology. The cross-sectional analysis demonstrates that the cultural politics of the Austrian extreme right ranges from appropriated national symbols to coded National Socialist iconography. These politics pervasively construct a gendered and racialized national body, policed by a ‘strict father’ and nurtured by a ‘self-sacrificing mother’, *vis-à-vis* an apocalyptic threat scenario identified with migration, intellectual and political elites, cosmopolitanism and progressive gender politics.

The research reported in this paper was conducted as part of a large-scale, longitudinal study of the construction of national identity/ies in Austria. The study, carried out within the framework of the Discourse Historical Approach (DHA), compiled a multifaceted and triangulated data set throughout 2015 and compared its results to similar constructions in 2005 and 1995.^1^ The core notions of the study, as in the previous studies, were: (1) the interdiscursively related construction of the so-called *Homo Austriacus/Homo Externus*; (2) the narration and construction of a shared political past; (3) a shared culture; (4) a shared political present and future; and (5) the discursive construction of a ‘national body’.^2^

We argue that it is the integration of the first and fifth categories—the *Homo Austriacus* and the national body—that characterizes the discourse of the far and extreme right in Austria, becoming the focal point of discursive practices and argumentative patterns, and serving related constructions of ‘us’ versus ‘them’. Such constructions emphasize *national uniqueness and (inner) homogeneity* and downplay *heterogeneity and difference* within the population. Simultaneously, they also focus on *differences to members of other nations*, often resorting to stereotyping and singularization. Here, we focus on the extreme-right elements within the far right, which includes both mainstream populist and extreme actors. Indeed, the Freiheitliche Partei Österreichs (FPÖ, Freedom Party of Austria) must be distinguished from other populist far-right parties, not only in some of its ideological positions but in terms of its history and ties to National Socialism.^3^

The ‘national body’ is constructed in terms of a nation’s territory, its ‘natural’ landscape as well as its built environment (including architecture, sites and material art), its institutions (such as the executive, legislative and judicial branches) and its national ‘heroes’ (in, for example, politics, the military, sports or arts).^4^ Previous studies have linked constructions of the national body to the symbols and practices of ‘banal nationalism’ as identified by Michael Billig: abstract and seemingly apolitical, naturalized figurations of the nation lend themselves particularly well to everyday nationalisms.^5^ At this theoretical juncture, we explore the link between the national body (that is, its salient constructions in banal nationalism) and the construction of a *Homo Nationalis* in more detail, in particular the *gender and body politics*.^6^ It is here that the extreme-right imaginary of the *Volk*—imagined as ideal male and female bodies, their roles and relationships—is articulated most clearly.

Extreme-right discourses have reinforced this link, once part and parcel of fascist (and National Socialist) discourses in the imaginary of the *Volkskörper*.^7^ A people, in this articulation, are a single body or family related by blood. It is on this conceptual level that our analysis engages with contemporary extreme-right discourses, tracing their gender and body politics through their articulations of the national body as a threatened and, indeed, compromised body that must be protected and restored. We here encounter constructions of a pan-German nationalist identity that go back to National Socialist ideology and before. This paper therefore begins by tracing the history of the extreme right in the Second Republic of Austria to provide the relevant historical context. We then analyse continuities and changes of extreme-right ideologies in the intertextual and interdiscursive relationships across genres and publics, most prominently in popular culture, which recontextualizes and propagates the extreme right’s ideal subject. To do so, we also analyse some of the offstage and onstage performances of the contemporary extreme right as well as the historical continuities with National Socialist rhetoric and imaginaries.

## The far and extreme right in Austria: tracing the history of the FPÖ

The history of the FPÖ dates back to the years after the Second World War, and the political situation created and supported by the four post-war Allied Powers until the *Staatsvertrag* (state treaty) of 1955. Between 1945 and 1955, the Allies supported the development of a bipolar political scene by encouraging (1) the cooperation of socialist-democratic movements on the left and the formation of what would later evolve into the Sozialdemokratische Partei Österreichs (SPÖ, Social Democratic Party of Austria); and (2) the merging of various right-wing, conservative and pro-clerical movements into the Österreichische Volkspartei (ÖVP, Austrian People’s Party). Simultaneously, the forerunner of the FPÖ, the Verband der Unabhängigen (VdU, Association of Independents) was formed by incorporating political movements comprised of ‘old Nazis, German nationalists and a fair number of liberals’,^8^ who were deliberately prevented from joining either of the two mainstream parties of the left and right.

In the 1949 parliamentary elections, the VdU won 12 per cent of the vote and soon called for ‘the abolition of all laws governing de-Nazification procedures’.^9^ Their argument implied that the real victims were not those persecuted by the Nazi regime but those who had profitted by acquiring Jewish property and former members of the NSDAP.^10^ The VdU thus used a ‘grotesque conception of fascism . . . to attack the de-Nazification policies of the government and to equate Nazism with other political systems’.^11^ Hence, the VdU allowed for a revival of Austrian ‘pro-fascist’ sentiment on a national scale and made it a significant element of the country’s political make-up in the coming years.

Following an internal crisis of the VdU, the FPÖ was established in 1955–6 ‘as a German nationalist party of the far right, in which former, seriously incriminated National Socialists took the leading functions’.^12^ Indeed, the first FPÖ leader, Anton Reintaller, had been a high-ranking member of the Austrian NSDAP and the SS, but also held the position of agriculture minister in the treacherous Austrian government of Seyß-Inquart in 1938. All this made the FPÖ the ‘successor to the Austrian NSDAP’ in all but name.^13^

When Friedrich Peter (another former SS member) took over the FPÖ in 1958, the party’s agenda did not change significantly. However, throughout the 1960s, the drive to moderate the FPÖ could be observed when both liberal and national views were given a voice. In 1970 the newly elected Chancellor Bruno Kreisky (SPÖ) formed a minority government with the support of the FPÖ and appointed ‘four former NSDAP members to ministerial posts’.^14^ This confirmed the FPÖ’s participation in mainstream Austrian politics, signalling ‘that the SPÖ, in order to gain power, could do business with former Nazis in a pragmatic way’.^15^ In 1986 the FPÖ’s national convention witnessed a coup led by Jörg Haider, who became the new chairman of the party. Haider’s rise marked the turn of the majority of the FPÖ to radical and nationalist/nativist views. Employing strongly antisemitic, anti-foreigner and revisionist slogans, Haider led the FPÖ to successful elections at all levels.

Throughout the late 1980s and early 1990s, the nationwide anti-foreigner petitions championed by Haider’s FPÖ, most prominently the 1992 ‘Austria First’ petition, significantly increased its electoral support.^16^ In the parliamentary elections of 1999, the FPÖ received an unprecedented 26.91 per cent of the vote and, for the first time in its history, took second place. After brief negotiations, the FPÖ signed a coalition agreement with the ÖVP and entered the federal government in early 2000. This was the first time an extreme-right party that had frequently expressed both coded and explicit praise for Nazism came to power in a European Union (EU) member state.^17^

Although sanctions against the Austrian government were eventually introduced by the fourteen other EU member states, it was not this external pressure but the controversial performance of the ÖVP-FPÖ government that pushed the FPÖ into gradual decline. At the next parliamentary elections, the FPÖ (headed by Heinz-Christian Strache, a former confidante of Haider) and the Bündnis Zukunft Österreich (BZÖ, Alliance for the Future of Austria), a splinter group headed by Haider, managed a combined 15.1 per cent of the vote. Following extensive negotiations, neither party became part of the new government, formed in early 2007 and headed by the SPÖ. And, thus, after eight years in government, the FPÖ returned to its opposition role until December 2017, when it became the junior partner in a governing conservative coalition between the ÖVP and FPÖ. After Haider’s death in 2008, the BZÖ dwindled into electoral insignificance. The fact that the FPÖ uses a vast array of populist strategies has sometimes obscured the fact that it continues to follow a xenophobic, anti-EU and anti-immigration agenda. The misogynist, anti-intellectual, anti-modern and anti-urban or anti-cosmopolitan aspects of its agenda have gone even more unnoticed. Indeed, today’s FPÖ has a ‘softer’ face in public, while mainstream parties have appropriated many policies formerly suggested by the FPÖ. In this way, the dominant agenda of the FPÖ has become normalized not only as part of the political mainstream,^18^ but also in popular culture.

## A cross-sectional analysis of the contemporary extreme right

**Figure 1:**
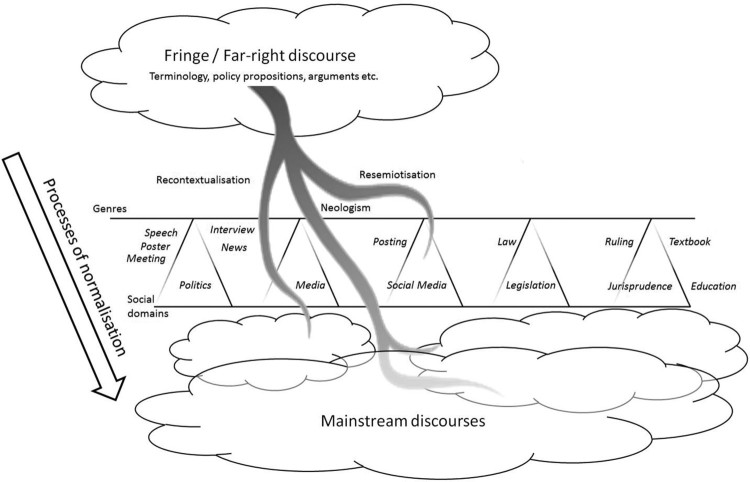
The normalization of far-right ideologies

The following analysis covers recent recontextualizations and semiotic reinterpretations (i.e. resemiotizations) of extreme-right ideology in Austria. Specifically, we provide a cross-sectional analysis of the fields of politics (party politics, part-affiliated organizations and media) and popular culture (music). This allows us to trace continuities not only historically but across fields, showing how extreme-right positions are recontextualized from closed-door meetings (that is, offstage politics^19^), unofficial handbooks and pamphlets to election campaigns (posters, speeches and television debates, that is, onstage politics) and ultimately normalized in popular culture. Our focus here is on the immensely popular musician Andreas Gabalier and how his songs, live performances, interviews and speeches as well as album covers construct the ideal subject of the extreme right. The advantage of such a cross-sectional approach is that it reveals the intertextual links between party politics and other discursive fields, sometimes evident and sometimes coded. This may be read as an appropriation of popular culture or an individual artist by party politics, but one might also regard it as the penetration of extreme-right ideology into seemingly innocuous entertainment as part of an ongoing process of *normalization* (see Figure 1). From the latter perspective, normalization describes how ideologies are incorporated into the mainstream—not only of politics but of popular culture and other fields as well—through recontextualizations and semiotic reinterpretations, usually moving from offstage to onstage, and across fields as well as genres.

## Recontextualizing extreme-right ideology: from closed-door meetings to handbooks

Arguably, no public figure represents extreme-right ideology within the FPÖ better than Johann Gudenus, head of the FPÖ’s Vienna chapter and Deputy Mayor of Vienna since 2015. Gudenus studied law in Vienna and Russian in Moscow, and also completed an M.A. at the Diplomatic Academy of Vienna. He maintains excellent relations with Russia, opposes the EU’s sanctions against it, and adopts an extremely sceptical stance towards the EU. Gudenus (whose father was convicted of Holocaust denial) is well known as one of the authors of the FPÖ’s programmatic agenda; moreover, his explicitly racist, nativist, antisemitic and homophobic offstage utterances have frequently been leaked to the press and caused scandals.

While addressing closed-door meetings, Gudenus has often expressed ideological positions in unambiguous terms. These have included racial policies supporting the purity of Europe: ‘Europe is the cradle of the white race. We demand a Europe-wide, coordinated policy for the family and reproduction, including a commitment to the fact that Europe is white.’^20^ They have also included the defamation of political opponents using antisemitic slurs: ‘If you mix red and green together, you get yellow. And yellow is the colour of Judas, it is the colour of treachery.’^21^ They have also included a denial of basic human rights (‘Asylum is not a human right’^22^) as well as homophobic, conspiracy theories of a doomed nation: ‘The powerful European lobby of homosexuals wants absolute equality for homosexuals and lesbians. It is hard to imagine where all this will lead.’^23^ Due to his high-ranking position in Austrian politics, Gudenus’s more public appearances directly recontextualize an offstage agenda into onstage performances and policies.

Gudenus’s utterances bear a strong similarity to the party-affiliated publication *Für ein freies Österreich* (For a Free Austria), written by Michael Howanietz, a local FPÖ politician.^24^ Although not officially party doctrine, the book closely mirrors the *Handbuch freiheitlicher Politik* (Handbook of FPÖ Politics), drafted by the party leadership to serve as an internal guide for party functionaries regarding key policy areas.^25^ Our focus here is on the former publication as it constitutes a less constrained articulation of extreme-right ideologies, free of the rhetorical limitations that even the FPÖ, as an established party, largely has to respect in its onstage politics.

*Für ein freies Österreich* is introduced by approving forewords written by the FPÖ’s chairman, Heinz-Christian Strache, and vice-chairman, Norbert Hofer. The text defines itself as a call for ‘an autonomous, independent country’, independent, that is, of transnational organizations, international law, international economy and the exchange of goods, all of which are seen as ways that Austria is controlled by others.^26^ Significantly, the various argumentative strands of the book—all ultimately intended to save Austria from immanent doom—are linked to the extreme right’s constructions of the national body; that is, the book calls for ‘an independent country that depends on its many existing strengths, its nature, its infrastructure and the productive power of its people’.^27^

The book relates this initial statement to arguments concerning the national body as ‘state territory’ and its ‘borders’, as ‘landscape and nature’, and as ‘the core family’ and ‘procreation’. Indeed, the call for ‘liberation’ from dependency is presented as a duty to ‘our children’, particularly that of men to their families: ‘We owe it to those who come after us, our children.’^28^ The book is also very clear about the link between identity and the nation; the nation, once the main carrier of identity, has been replaced by societies and ‘clubs’ and ‘brands’, ‘weak prosthetics’ for the true belonging of national identity.^29^ Such true belonging or *Heimat* supposedly still exists in the country and rural areas, manifest in higher birth rates, manual labour as in centuries past, hardy craftsmen and ‘timeless’ values.^30^ In this context, Howanietz emphasizes the spiritual and biological link of a people to the soil, the ‘most sacred property of the community’, namely, the nation.^31^ Equating soil and blood, to protect this eternal *Heimat* is thus to protect one’s true self.^32^

In the extreme-right world view articulated in the book, which casts the modern and in particular the urban and intellectual as weak as opposed to the archaic, migrants are a threat precisely because they have stronger identities: their ‘assault’ or slow invasion to ‘demographically displace’ the Austrian people makes the latter ‘a species on the brink of extinction’.^33^ To be modern, to include women in the workforce and so on is thus seen as a form of ‘self-extinction’ or ‘self-destruction’,^34^ echoing Thilo Sarrazin’s *Deutschland schafft sich ab* (Germany Is Doing Away with Itself).^35^ The battlefronts of this struggle are many: ‘It starts with a few English terms, inappropriate concessions to culturally foreign (*kulturfremde*) “neo-Austrians” and years with a low birth-rate. Every unborn potential mother and father of the future accelerates the process of self-annihilation.’^36^ This, of course, links directly to attacks on legal abortion (see below) and is elaborated in the FPÖ’s *Handbuch*. Here, the argument is presented in the form of statistics: ‘in 2009, 76,344 births and 50,000 abortions killed some 125,000 children [meaning that] 4 out of 10 children are killed in the womb. This would make the uterus the place with the highest mortality rate in our country.’^37^ This not only constitutes an attack on women’s legal right to seek an abortion—up until three months after conception, and beyond if there is grave threat to the physical or mental health of the pregnant woman or a serious risk that the child will be severely handicapped or if the pregnant woman was under the age of fourteen at the time of conception^38^—it also constructs any such woman as ‘killing a child’.

The gravest threat to the nation, however, is identified in decaying national pride: honour and loyalty to the community of the nation are seen as the foundation for loyalty and faithfulness in heterosexual relationships.^39^ This makes those who would weaken nationalism also traitors to the family: *die Familienzerstörer*, destroyers of families.^40^ This, ultimately, defines the core of the FPÖ’s current gender politics as a deeply conservative biopolitics:

The child needs the safety of the family. Its pillars are father and mother as positive male and female example. Both have been made deeply insecure in their self-understanding by the deliberate demolition of their nature-given roles. Their disorientation leads to temporary relationships, because the image corresponding to their inner longing for the respective counterpart is not found. Man, who has been cast from his throne at the head of the family, still longs for a female partner who, in spite of the girls-own-the-world magazines, is still able to think in homemaking categories, whose maternal drive exceeds the imposed ambitions for personal fulfilment. Woman, redefined by feminist deconstructionist ambitions as a birth-certificate mother required to pursue personal fulfilment, still longs for a real man who gives her all the emotional and economic security that a young mother needs to devote herself almost free from worry to her offspring.^41^

The book shamelessly uses hyperbole and straw-man fallacies to drive home this point: ‘Because we are still permitted, without official permission, to have children and raise them as best we can, independent of ideological approaches that want to tear children away from their parents immediately after birth.’^42^ The purported conspiracy to brainwash children and ‘abolish natural genders’ is seen as the reason for women wanting a career and financial independence, which in turn is seen as the reason for ‘many young women misrepresenting an initially desired pregnancy as sexist harassment’,^43^ and ignoring their ‘maternal instincts’.^44^

The other side of this gender politics is to denounce ‘effeminate’ and ‘feminized’ modern men, biologically destined to be ‘provider and protector of the family’.^45^ Alternative gender constructions are cast as a leftist conspiracy to undermine masculine ideals: ‘Sportsmen are the last remaining idols who can still be regarded as “heroes”, since other traditional ideals, such as the embodiment of military virtue, the principles of chivalry, all had to be sacrificed to the Zeitgeist.’^46^ Blame for this decay of traditional gender roles is placed on the left, feminists, civil society, non-governmental organizations (NGOs), international organizations, corporations and, most of all, the media, which the book describes as ‘weapons of mass destruction’ when it comes to the destruction of the *Volk*. ‘Fake news’ (and the German terms *Lügenpress* or *Systempresse*, heavily used in Nazi propaganda to discredit first the Weimar and then international press^47^) is a core notion of the contemporary extreme right in Austria.

Comparing this internal weakness to the external threat identified as migrants, the book offers two alternative prognoses for the future: the true Austrian *Volk* will either slowly degenerate and die off, ‘eaten from the inside like wasp larvae eat maggots’,^48^ or current developments will lead to a violent ‘civil war’.^49^ The author clearly prefers the latter, arguing that like any conflict it would be ‘productive’ and ‘awaken potential’.^50^ Either way, he concludes, ‘Europe will burn’.^51^

## The extreme right in election campaigns

**Figure 2:**
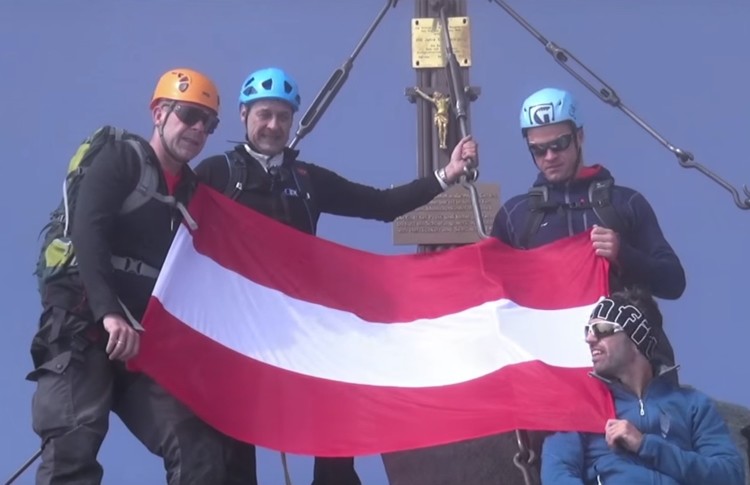
Vilimsky (Party Secretary and MEP), Strache, Gudenus and Kickl (former party Secretary, now Home Secretary) brandishing the Austrian flag on top of Großglockner (Austria’s highest mountain)

**Figure 3:**
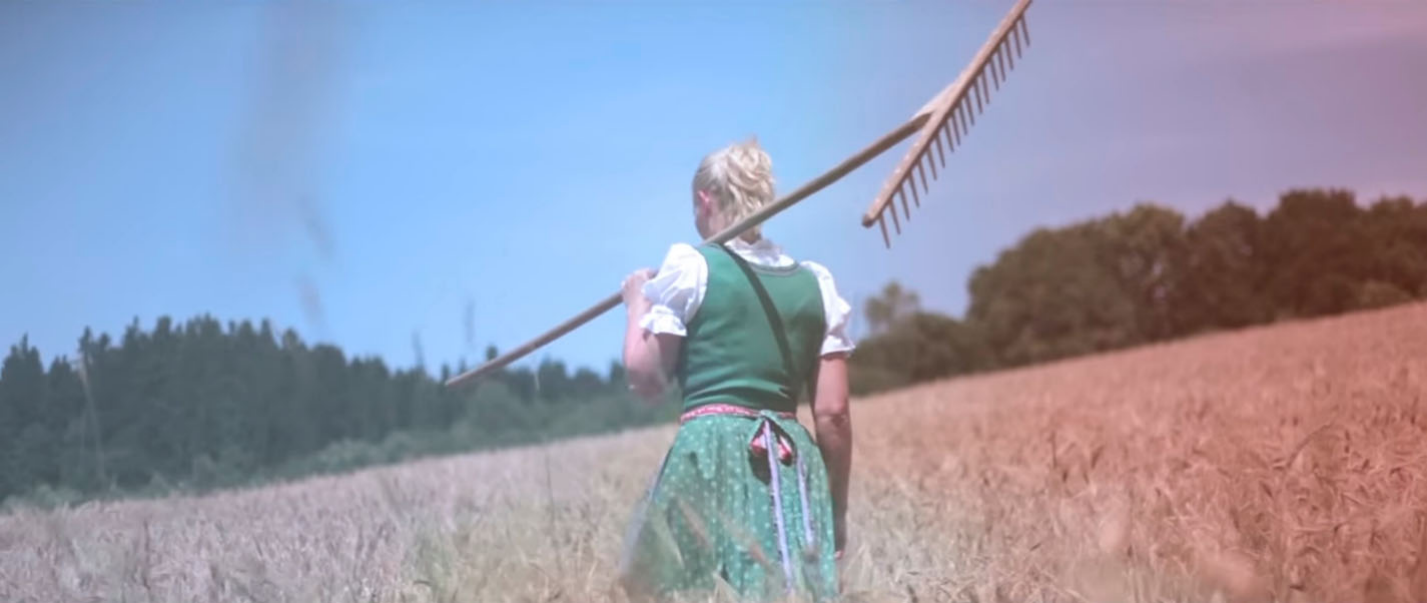
Symbolic image of The Nation^53^

Since Heinz-Christian Strache took control of the FPÖ in 2005, the public activities of the party—particularly in election campaigns and social media—have seen a softening of extreme-right positions and an increase of banal nationalism:^52^ displaying the Austrian flag; singing the national anthem; and showing an abundance of other symbols of national pride. In terms of national identity, these symbols are linked to deeply conservative constructions of the national body, such as pristine landscapes, snow-covered mountain tops, skiing, traditional agriculture and farmers, and religious symbols of Christianity. In many instances, the respective texts and performances feature Strache himself wielding these symbols (see Figures 2 and 3).

Since these are symbols of the nation rather than the party, their recontextualization and semiotic reinterpretation by the FPÖ constitute a provocative appropriation linked to the party’s claim to be the only one that represents ‘the people’ and ‘the nation’ or *Heimat*. Indeed, with Strache’s leadership came a rebranding of the FPÖ as the Soziale Heimatpartei, the Social Homeland Party (a label it shares with the extreme-right Nationaldemokratische Partei Deutschlands (NPD) of Germany). Two such provocations relate to Austria’s national anthem: first, the public refusal to sing the amended national anthem (since 2011, the lyrics include ‘daughters’ alongside ‘sons’), thus breaking the relevant law; and, second, the use of an alternative anthem, titled ‘Immer wieder Österreich’, for the party’s campaigns. The lyrics of this song incorporate a well-known chant by football fans at matches played by the Austrian national team—‘Immer wieder, immer wieder, immer wieder Österreich’ (‘Time and again, time and again, time and again Austria’)—but also appeal to the ‘honesty, righteousness and loyalty’ of all true Austrians, calling on them to ‘pledge’ their loyalty to ‘their country’. The song features in a campaign video that semiotically reinterprets the lyrics of the actual Austrian anthem and alludes to the aesthetics of Nazi-era films (such as Leni Riefenstahl’s work), that focus on mountains, streams and other aspects of the national body, white men and women, industry in the form of hammers, and agriculture in the form of fields (see Figure 3).

**Figure 4:**
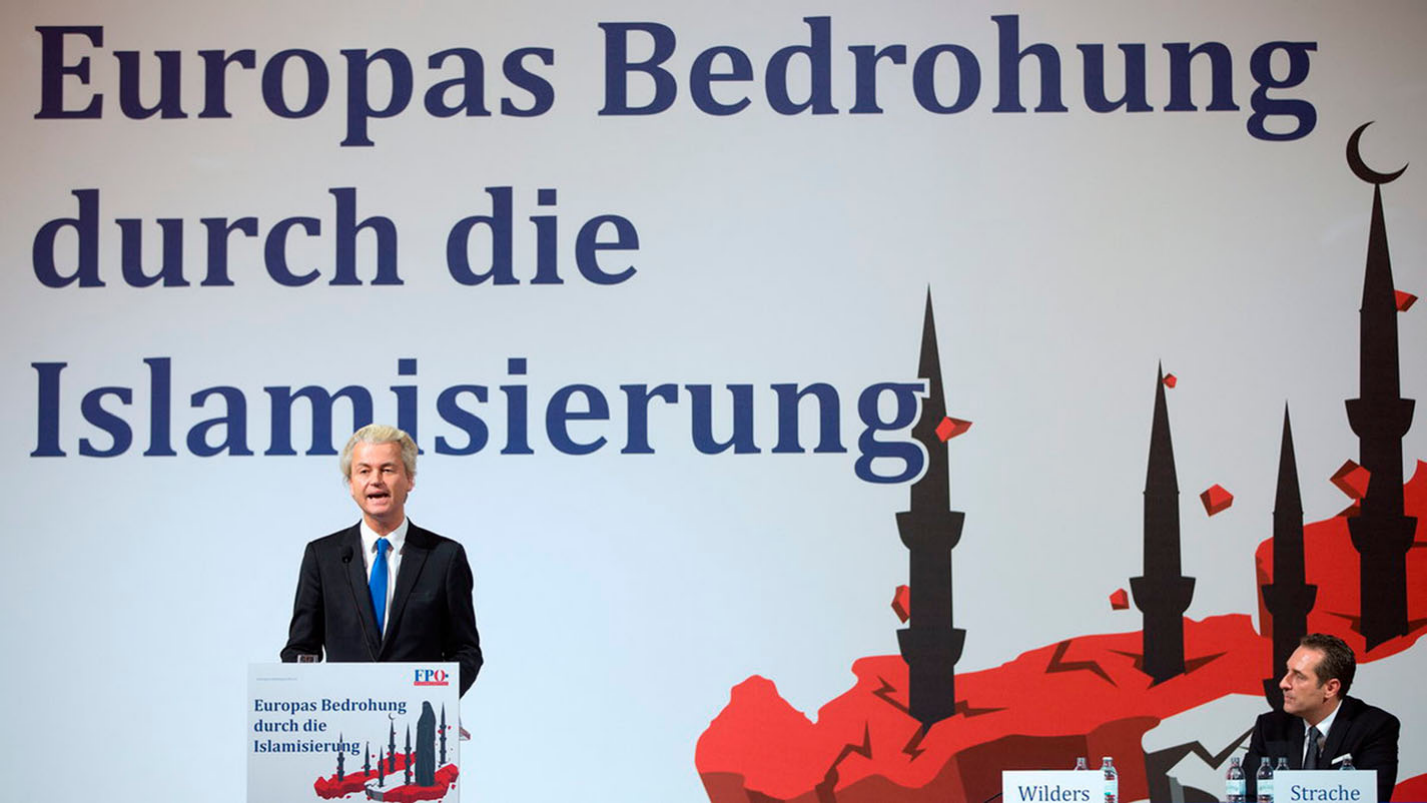
The wounded national body: Geert Wilders visits the FPÖ (‘Europe’s threat from Islamization’)^56^

Further notable provocations pertaining to the national body involve the use of religious imagery and symbols as well as the redefinition of religious concepts (such as *Nächstenliebe*, ‘neighbourly love’ or ‘charity’). The Catholic Church has protested against the party’s accompanying claims to represent (and defend) the Christian heritage of Austria in the face of an alleged Islamic invasion. Indeed, the Austrian far and extreme right’s Othering has come to focus strongly on religion, that is, Islam. In this opposition, the Self is largely culturalized (Christian-Judean culture) and may even be secularized (European Enlightenment) or Europeanized (European values), whereas the Other is cast as an ethnic Other, mediaeval/pre-modern/barbaric or religious zealot/fanatic or terrorist threat.^54^ The following are but two examples of the links constructed between religious imagery and the national body: (1) at a rally during the 2015 Vienna election campaign, Gudenus described the ringing of the Pummerin, the prominent church bell of Vienna’s St Stephen’s cathedral, as the ‘heartbeat of Austria’ (8 October 2015); (2) a poster displayed at the Dutch right-wing populist Geert Wilders’s visit to the FPÖ visualized this threat as the violated national body, coded in the colours of the flag (red–white–red) and wounded by the spear-like minarets of mosques (see Figure 4).^55^

Such discursive strategies for anthropomorphizing, culturalizing and ethnicizing the national body go back many years. Indeed, the onstage politics of the extreme right in Austria are remarkably culturalist and biologistic.^57^ Such constructions depend on emphasizing the internal homogeneity of the Austrian people or *Volk*, while highlighting difference to other nations and peoples.

Despite the focus on ‘Islamization’—and despite the fallacious claim that the FPÖ is ‘the new Jew’ or being persecuted (part of an ongoing strategy to present itself as the victim of the political establishment and mainstream media^58^)—antisemitism remains part of extreme-right discourse. While this is usually coded (such as ‘the East Coast’, meaning the *New York Times* and supposedly powerful Jewish lobbies on the East Coast of the United States) in public, there are still prominent exceptions, such as the so-called ‘Facebook incident’ of 2012.^59^ An even more prominent case is Barbara Rosenkranz, former MP and FPÖ candidate for president in 2010, who implicitly denied the Holocaust during her campaign.^60^ Rosenkranz also published a book that lashed out against feminism and gender mainstreaming.^61^ She vocally opposes civil partnerships for homosexual couples because—she argues—the legal definition of marriage includes the intention to have and raise children.

In the 2016 presidential election, the FPÖ candidate Norbert Hofer’s campaign offered a toned-down version of such ideology. The campaign was organized around central notions connected to the national body and its protection: *Heimat* (homeland); *Heimatliebe* (love for the homeland); *Sicherheit* (security); *Flagge zeigen* (literally ‘to show flag’, German idiom for ‘to show one’s colours’). Indeed, the Austrian flag and its colours were dominant in his campaign (some posters show as many as five Austrian flags in different shapes and sizes), but it also made use of religion and religious imagery.

Despite notable moderation, the FPÖ’s gender politics remain patriarchal. In several television debates leading up to the election, Hofer maintained that any marriage of gay or lesbian couples as well as related adoption rights would destroy ‘natural family structures’: ‘The life partnership of man and woman becomes a family only through the child. Those who raise a child on their own create a family with the children.’ He also voiced his rejection of gender mainstreaming: ‘The aim of “gender mainstreaming” is nothing short of creating the ‘new human being’ that Marxists-Leninists already aspired to.’ Similar to many fundamentalist Tea Party Republicans in the United States, Hofer also rejected ‘pro-choice’ policies for women, that is, women’s right to decide on abortion.^62^

In this context, Hofer also described ‘the womb as the place with the highest mortality rate in our country’.^63^ This gendered discourse clearly attempts to govern and regulate women’s bodies and minds, thus objectifying and disciplining women in a way that is characteristic of the extreme right. In the gender politics of such ideologies, the ‘national family’ must preserve the traditional paternalistic order of the sexes and maintain the nation’s body as white and pure (see Gudenus’s statements earlier). This draws on conservative and fascist imaginaries as extensively investigated by Andreas Musolff and John Richardson in their research on the concept of the *Volk* and the *Volkskörper* across German and British nationalist writing since the eighteenth century.^64^ In summary, the extreme right’s salient construction of the national body and use of associated symbols shows a constant effort to mobilize feelings of national pride but equally of national emergency, of threat and the need to defend but also to reassert gender relations as heterosexual and primarily reproductive.

## The extreme right in popular culture

The Austrian brand of banal nationalism—in the form not only of state symbols, such as the anthem or flag, but mythopoeia that enshrines mountain tops, farmers and rural villages as the heartland of the Austrian people—is well established in collective identity. Popular culture, including popular music in its pop, folk or *Schlager* variants, often draws on such images, sometimes naively and sometimes with a pinch of irony. Figurations of banal nationalism are nothing new in pop songs such as ‘I Am from Austria’ by Reinhard Fendrich (earnest in its nostalgic sense of national belonging) or ‘Skifoan’ by Wolfgang Ambros (ironic in its exaggerations). There is also a history, extending well beyond Austrian borders, of extreme-right discourse in songs,^65^ including contemporary rock/popular music.^66^

A recent development, however, is the particular brand of pop music performed since 2008 by Andreas Gabalier, which he himself has labelled ‘Volks-Rock’n’Roll’. In his music, lyrics, stage performances and interviews, Gabalier not only blurs the boundaries of musical genres, he also realizes the culturalization of extreme-right ideologies on a grand scale. He is currently the most popular performing artist from Austria, playing concerts for audiences of 70,000; he has sold more than two million records and attracts young audiences from rural and urban areas alike.

**Figure 5:**
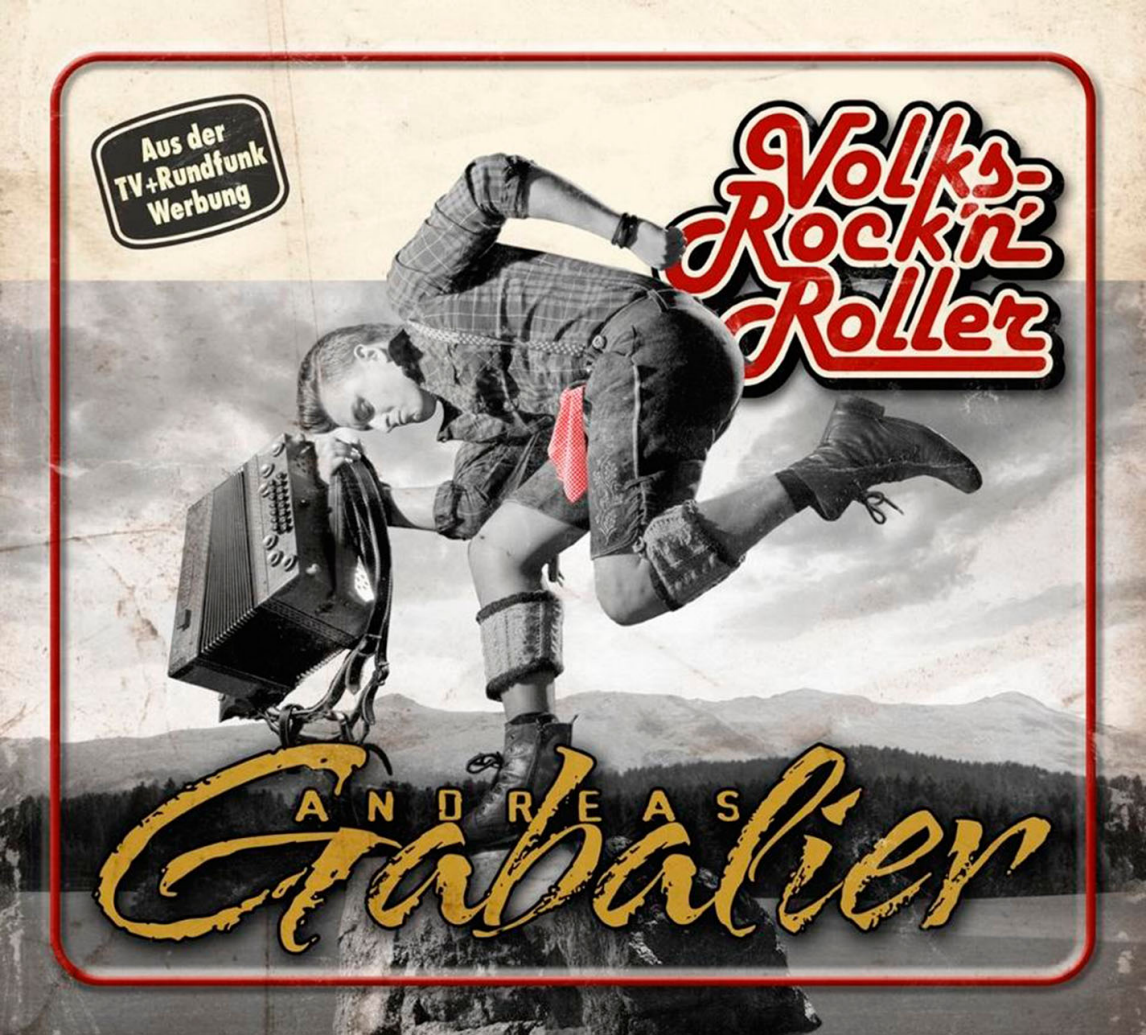
Cover of Andreas Gabalier’s album *Volks-Rock’n’Roller* (Koch Universal Music 2011)

The political in his performances ranges from forms of banal nationalism and a deeply conservative view of *Heimat*, to a disconcertingly chauvinistic view of gender roles and the family, as well as ambiguous language and visuals that may well be read as coded allusions to National Socialism. The latter has caused some mild scandal in Austrian media, most prominently with respect to the controversial cover of his album *Volks-Rock’n’Roller*, which depicts Gabalier in an oddly contorted posture intimating the outlines of a swastika (see Figure 5).^67^ Among other codes, his songs contain references to the special friendship between Germans, Austrians, Italians and Japanese,^68^ as well as the friendship between *Kameraden* (comrades, usually in arms) that he describes as an ‘Eisernes Kreuz’ (Iron Cross), defying storms and fire on a mountain top.^69^

Gabalier’s popular music is not, however, always as carefully hedged and coded with regard to its extreme-right politics. The 2015 song ‘Königin der Alpen’ (Queen of the Alps) describes Gabalier as a ‘Germanic prince’,^70^ indicating pan-German nationalist convictions that both predate and outlive National Socialism. Holding that Austria culturally (and, at least by implication, also politically) is part of the Germanic nation,^71^ this view is closely tied to assumptions of cultural and racial supremacy.^72^

More overtly, Gabalier’s music has followed the traditional formula of romantic love, family and homeland. His songs frequently name specific regions, mountains, valleys or rivers, describing their beauty. In this scenery, Gabalier positions himself not only as an advocate but also a representative of traditional masculinity *vis-à-vis* an equally traditional femininity. In songs like ‘Meine Heimat’ (My Homeland) he presents these as values passed on to him by his grandfather, connecting him to the land and his ancestors ‘forever’. One of the most important attributes of the thereby constructed homeland is a place ‘where our girls love us because they know what kind of men we are’.^73^ The traditional heterosexual matrix is thus inscribed into the national body. In many ways, the song ‘Bergbauernbuam’ (Mountain Farmer Boys) popularizes the ideal subject of the extreme right: a hard-working mountain farmer, in close communion with nature, a man carved from the wood of local trees, strong and virile as a bull.^74^ This ideal man lives in an archaic time when the man of the house has to leave the homestead and venture into the ‘wide world’ to provide for his ‘little girl’, indexing the pervasive misogyny of the extreme right’s gender politics.

Public controversy around Gabalier’s work—primarily in interviews and television debates—has led him to take an even clearer stance in more recent songs. In ‘Mei Großvater hat gesagt’ (My Grandfather Said), Gabalier praises traditional, girlish femininity in familiar terms but ends with a remark on progressive, emancipated women. He links the lack of femininity of emancipated women to ‘being like a man’, and takes the fact that this does not sexually arouse him as proof of his own heterosexuality *vis-à-vis* the purported sexual confusion of other, lesser men:

Now I would almost have forgotten, there is that kind of woman too
Who builds on anything but femininity
It flatters us, but it doesn’t turn us on
Why does girl today have to be like a man?
Completely grim, almost in pain from emancipation
So that you lose all joy in making out
But not all of us are into men
We much prefer to nibble at a real girl.^75^

The song’s phrasing in the final two lines not only reveals anxiety *vis-à-vis* an (imagined) homosexual majority and defines who is to be regarded as a true girl, denying emancipated women that gender identity, but it also incorporates the victim–perpetrator reversal that Gabalier publicly expressed in interviews and at the ceremony for the Amadeus Music Award, Austria’s largest music award. The true heterosexual man, he implies, is in the minority today and must defend his natural right not only to desire girls but to demand that girls be desirable for him.

## The idealized body under threat

Our conceptual focus on identity politics as articulated in body and gender politics, theorized through the notion of the *national body*, facilitates a clear view of the extreme right’s imaginary of a *Volkskörper* in its contemporary forms. This imaginary reveals National Socialist ideology to be in evidence not only superficially (for example, in imagery or symbols, used principally to provoke), but as a politics of heteronormative, procreational gender roles predicated on the ideal of national integrity as racial purity. The ideal *Homo nationalis* is thus constructed as white, binary (male/female), heterosexual, (culturally) Christian and rooted (by blood) in the soil of the national body. This ideal is opposed to a negative imaginary that constructs Austrians as compromised on all levels, colonized by ‘parasites’ as well as ruled by (foreign) elites, incapable of action or emancipation. The ideal subject of the extreme right, our analyses show, is constructed across a number of social fields and contexts, evidencing continuities from offstage politics to popular culture.

With these results in mind, we must acknowledge that the FPÖ, as the only established party in Austria that has consistently provided a home for extreme-right positions, is far from homogeneous. The ideological positions articulated by its leading figures range from extreme-right and pan-German nationalism to toned-down positions, particularly in their public iterations. Indeed, the ability to alternate between ‘strong’ and ‘soft’ performances according to context and audience is a defining characteristic of the contemporary far right in Austria. Both allusions and encoded references to extreme-right and Nazi ideologies are thus part of the strategy of calculated ambivalence that ensures deniability. Closely related to these strategic performances are processes of normalization, removing taboos through recontextualization and semiotic reinterpretations as aspects of extreme-right imaginaries move from offstage to onstage and from party politics to popular culture.

In this dynamic, we would argue, the counterpart to the ideal extreme-right subject must not be overlooked. It is the discursive construction of a multilayered and ever-present threat to the nation and the ideal subject identified with that nation; in as much as this threat is extreme, the defence is justified to be extreme as well. This is an imaginary of perpetual crisis, reminiscent of Orwell’s perpetual and omnipresent (yet invisible) war in *1984*; likewise, it is invoked to mobilize voters and stifle voices of criticism as ‘unpatriotic’ in the face of imminent extinction. Its culturalization has made it more pervasive, more naturalized and seemingly less political than ever before.

## Footnotes

1 Ruth Wodak, Rudolf de Cillia, Martin Reisigl and Karin Liebhart, *The Discursive Construction of National Identities*, 2nd edn (Edinburgh: Edinburgh University Press 2009).

2 Ibid., 30.

3 Leading members of the party have been investigated for and convicted of activities such as denying the Holocaust or violating the *Verbotsgesetz* or Prohibition Act of 1947, which banned the Nazi Party and aimed to suppress any potential revival of Nazism; see works such as Jakob Engel and Ruth Wodak, ‘“Calculated ambivalence” and Holocaust denial in Austria’, in Ruth Wodak and John E. Richardson (eds), *Analysing Fascist Discourse: European Fascism in Talk and Text* (London and New York: Routledge 2013), 73–96.

4 Wodak, de Cillia, Reisigl and Liebhart, *The Discursive Construction of National Identities*; Rudolf de Cillia and Ruth Wodak (eds), *Gedenken im ‘Gedankenjahr’: Zur diskursiven Konstruktion österreichischer Identitäten im Jubiläumsjahr 2005* (Vienna: Studienverlag 2009).

5 Michael Billig, *Banal Nationalism* (London, Thousand Oaks, CA and New Delhi: Sage 1995).

6 For a discussion of biopolitics and reproductive politics of the European far right, see Jemima Repo, ‘Thanatopolitics or biopolitics? Diagnosing the racial and sexual politics of the European far-right’, *Contemporary Political Theory*, vol. 15, no. 1, 2016, 110–18.

7 Andreas Musolff, *Metaphor, Nation and the Holocaust: The Concept of the Body Politic* (London and New York: Routledge 2010); Ruth Wodak, *The Politics of Fear: What Right-Wing Populist Discourses Mean* (London, Thousand Oaks, CA, New Delhi and Singapore: Sage 2015).

8 Heribert Schiedel and Wolfgang Neugebauer, ‘Jörg Haider, die FPÖ und der Antisemitismus’, in Anton Pelinka and Ruth Wodak (eds), ‘*Dreck am Stecken’: Politik der Ausgrenzung* (Vienna: Czernin 2002), 11–31 (16). All translations from the German, unless otherwise stated, are by the authors.

9 Walter Manoschek, ‘FPÖ, ÖVP, and Austria’s Nazi past’, in Ruth Wodak and Anton Pelinka (eds), *The Haider Phenomenon in Austria* (New Brunswick, NJ and London: Transaction 2009), 3–15 (5).

10 Michał Krzyżanowski and Ruth Wodak, *The Politics of Exclusion: Debating Migration in Austria* (New Brunswick, NJ: Transaction 2009).

11 Schiedel and Neugebauer, ‘Jörg Haider, die FPÖ und der Antisemitismus’, 15.

12 Ibid., 16.

13 Manoschek, ‘FPÖ, ÖVP, and Austria’s Nazi Past’, 6–7 .

14 Schiedel and Neugebauer, ‘Jörg Haider, die FPÖ und der Antisemitismus’, 17.

15 Ruth Wodak, Johanna Pelikan, Peter Nowak, Helmut Gruber, Rudolf de Cillia and Richard Mitten, *‘Wir sind alle unschuldige Täter!’ Diskurshistorische Studien zum Nachkriegsantisemitismus* (Frankfurt am Main: Suhrkamp 1990), 290; Wodak and Richardson (eds), *Analysing Fascist Discourse*.

16 Martin Reisigl and Ruth Wodak, ‘“Austria first”: a discourse-historical analysis of the Austrian “anti-foreigner petition” in 1992 and 1993’, in Martin Reisigl and Ruth Wodak (eds), *The Semiotics of Racism: Approaches in Critical Discourse Analysis* (Vienna: Passagen 2000), 269–303.

17 Wodak, *The Politics of Fear*.

18 Markus Rheindorf and Ruth Wodak, ‘Borders, fences, and limits—protecting Austria from refugees: metadiscursive negotiation of meaning in the current refugee crisis’, *Journal of Immigrant & Refugee Studies*, special issue on ‘Mediatization and Politicization of the Refugee Crisis’, vol. 16, no. 1–2, 2018, 15–38.

19 Ruth Wodak, ‘Political discourse analysis—distinguishing frontstage and backstage contexts: a discourse-historical approach’, in John Flowerdew (ed.), *Discourse in Context*, Contemporary Applied Linguistics, vol. 3 (London and New York: Bloomsbury 2014), 321–45.

20 Leaflet by the FPÖ’s youth organization, Ring Freiheitlicher Jugend*,* under the chairmanship of Johann Gudenus.

21 Johann Gudenus at a rally in 2011 (reported in the FPÖ newspaper *Neue Freie Zeitung*, 27 October 2011).

22 ‘FP-Gudenus: “Stadt der Menschenrechte”—nicht mehr als ein schönes Schlagwort’, press release by Johann Gudenus, 19 December 2014, available on the *APA-OTS* website at www.ots.at/presseaussendung/OTS_20141219_OTS0076 (viewed 20 May 2019).

23 Speech by Johann Gudenus, Moscow, 11 September 2014.

24 Michael Howanietz, *Für ein freies Österreich: Souveränität als Zukunftsmodell*, ed. Norbert Hofer (Vienna: Freiheitlicher Parlamentsklub 2013).

25 *Handbuch freiheitlicher Politik*, 4th edn (Vienna: FPÖ-Bildungsinstitut 2013).

26 Heinz-Christian Strache, ‘Vorwort’, in ibid., 6.

27 Ibid., 7.

28 Ibid.

29 Howanietz, *Für ein freies Österreich*, 15.

30 Ibid., 77.

31 Ibid., 141.

32 Ibid., 137.

33 Ibid., 19–20.

34 Ibid., 21.

35 Thilo Sarrazin, *Deutschland schafft sich ab: Wie wir unser Land aufs Spiel setzen* (Munich: Deutsche Verlags-Anstalt 2010).

36 Howanietz, *Für ein freies Österreich*, 20.

37 *Handbuch freiheitlicher Politik*, 4th edn (Vienna: FPÖ-Bildungsinstitut 2013), 160.

38 § 97 of the *Strafgesetzbuch* (StGB, Austrian Criminal Code), available on *jusline.at* at www.jusline.at/gesetz/stgb/paragraf/97; see also Christian Fiala, ‘Abbruch in Österreich: Schwangerschaftsabbruch in Theorie und Praxis’, available on *abtreibung.at* at http://abtreibung.at/fur-fachkrafte/hintergrundinformationen/abbruch-in-osterreich (both viewed 17 May 2019).

39 Howanietz, *Für ein freies Österreich*, 30–1; ‘Ehre’ and ‘Treue’, the two concepts the book praises in this context, formed the core of the SS motto ‘Meine Ehre heißt Treue’, which is banned under the Austrian law.

40 Ibid., 31.

41 Ibid., 32.

42 Ibid., 34.

43 Ibid., 118 and 110–12.

44 Ibid., 119.

45 Ibid.

46 Ibid., 35.

47 Cornelia Schmitz-Berning, *Vokabular des Nationalsozialismus* (Berlin and New York: Walter de Gruyter 1998), 327. For the current use of the notion, see Wolfgang Frindte and Nico Dietrich (eds), *Muslime, Flüchtlinge und Pegida: Sozialpsychologische und Kommunikationswissenschaftliche Studien in Zeiten globaler Bedrohungen* (Wiesbaden: Springer VS 2017).

48 Howanietz, *Für ein freies Österreich*, 117.

49 Ibid., 121.

50 Ibid., 133.

51 Ibid., 134.

52 Billig, *Banal Nationalism*.

53 Figures 2 and 3 are stills from ‘Immer wieder Österreich–Flagge zeigen’ (Time and Again Austria–Show Your Colours), FPÖ TV presidential election campaign video featuring FPÖ anthem, 8 October 2016, available on *YouTube* at www.youtube.com/watch?v=IcV6aUw4xhU (viewed 5 May 2019).

54 Ruth Wodak, ‘Integration and culture: from “communicative competence” to “competence in plurality”’, in Rainer Bauböck and Milena Tripkovic (eds), *The Integration of Migrants and Refugees: An EUI Forum on Migration, Citizenship and Demography* (Florence: European University Institute, Robert Schuman Centre for Advanced Studies 2017), 116–37; Wodak, *The Politics of Fear*, 125–90.

55 A recontextualization of an infamous Swiss People’s Party poster; see Hans-Georg Betz, ‘Mosques, minarets, burqas and other essential threats: the populist right’s campaign against Islam in Western Europe’, in Ruth Wodak, Majid KhosraviNik and Brigitte Mral (eds), *Right-Wing Populism in Europe: Politics and Discourse* (London and New York: Bloomsbury 2013), 71–88; and Aristotle Kallis, ‘Breaking taboos and “mainstreaming the extreme”: the debates on restricting Islamic symbols in contemporary Europe’, in Wodak, KhosraviNik and Mral (eds), *Right-Wing Populism in Europe*, 55–70.

56 Reproduced from the Austrian daily newspaper *Kurier*, 27 July 2015, available on the *Kurier* website at https://kurier.at/chronik/wien/staatsanwalt-ermittelt-gegen-rechtspopulist-geert-wilders/143.733.765 (viewed 8 May 2019).

57 Katharina Köhler and Ruth Wodak, ‘Mitbürger, Fremde und “echte Wiener”—Ein- und Ausgrenzungen über Sprache: Diskursive Konstruktion von Macht und Ungleichheit am Beispiel des Wiener Wahlkampfes 2010’, *Deutschunterricht*, vol. 63, no. 6, 2011, 64–74; Wodak, *The Politics of Fear*.

58 Karin Stögner, ‘Secondary antisemitism, the economic crisis and the construction of national identity in the Austrian print media’, *Critical Sociology*, vol. 44, no. 4–5, 2018, 719–32.

59 Wodak, *The Politics of Fear*.

60 Engel and Wodak, ‘“Calculated ambivalence” and Holocaust denial in Austria’.

61 Barbara Rosenkranz, *MenschInnen: Gender Mainstreaming—Auf dem Weg zum geschlechtslosen Menschen* (Graz: Ares 2008).

62 Television debate hosted by the Austrian television channel ATV, 8 May 2016.

63 Ibid.

64 Musolff, *Metaphor, Nation and the Holocaust*; John E. Richardson, ‘Fascist discourse’, in John Flowerdew and John E. Richardson (eds), *The Routledge Handbook of Critical Discourse Studies* (London and New York: Routledge 2018), 447–61.

65 David Machin and John E. Richardson, ‘Discourses of unity and purpose in the sounds of fascist music: a multimodal approach’, *Critical Discourse Studies*, vol. 9, no. 4, 2012, 329–45.

66 Anton Shekhovstov, ‘European far-right music and its enemies’, in Wodak and Richardson (eds), *Analysing Fascist Discourse*, 277–96.

67 Gabalier has denied such allusions and describes himself as the victim of a defamation campaign by the left. He has recently sued the director of the Viennese Konzerthaus (concert hall) for publicly stating that he would not allow Gabalier to perform at the venue due to the ideology he represents.

68 Andreas Gabalier, ‘Biker’, in Andreas Gabalier, *Herzwerk* (Koch Universal Music 2010).

69 Andreas Gabalier, ‘Mein Bergkamerad’, in Andreas Gabalier, *Herzwerk* (Koch Universal Music 2010). The Iron Cross was a military decoration in the Kingdom of Prussia and later, with a swastika added at its centre, in Nazi Germany.

70 Andreas Gabalier, ‘Königin der Alpen’, in Andreas Gabalier, *Mountain Man* (Koch Universal Music 2015).

71 Rudolf de Cillia and Ruth Wodak, *Ist Österreich ein ‘deutsches’ Land? Sprachenpolitik und Identität in der Zweiten Republik* (Innsbruck: StudienVerlag 2006).

72 Martin Reisigl and Ruth Wodak, *Discourse and Discrimination: Rhetorics of Racism and Antisemitism* (London and New York: Routledge 2001).

73 Andreas Gabalier, ‘Meine Heimat’, in Andreas Gabalier, *Da komm’ ich her* (Koch Universal Music 2009).

74 Andreas Gabalier, ‘Bergbauernbuam’, in Andreas Gabalier, *Herzwerk* (Koch Universal Music 2010).

75 Andreas Gabalier, ‘Mei Großvater hat gesagt’, in Andreas Gabalier, *Mountain Man* (Koch Universal Music 2015).

